# Beyond T-cell subsets: stemness and adaptation redefining immunity and immunotherapy

**DOI:** 10.1038/s41423-025-01321-7

**Published:** 2025-07-10

**Authors:** Dawei Zou, Xian C. Li, Wenhao Chen

**Affiliations:** 1 Immunobiology & Transplant Science Center, Department of Surgery, Houston Methodist Research Institute, Houston Methodist Hospital, Houston, TX USA; 2 Department of Surgery, Weill Cornell Medicine, Cornell University, New York, NY USA

**Keywords:** Stem-like T cells, Effector differentiation, CD4^+^ T cells, Cancer immunotherapy, Autoimmunity, Transplant rejection, CD4-positive T cells, Tumour immunology, Autoimmunity

## Abstract

T cells often acquire diverse phenotypes and functional states following activation. CD4^+^ T cells are traditionally classified into distinct effector subsets, such as Th1, Th2, Th17, and Tfh, on the basis of their cytokine profiles and functional roles. While this framework has advanced our understanding of adaptive immunity, it has limitations in explaining the persistence of T-cell responses in settings of autoimmunity and transplant rejection, in contrast to its limited efficacy in cancer. Moving beyond this subset-based framework, recent studies have revealed that stemness and adaptation are fundamental to CD4^+^ T-cell fate and function. Central to this new understanding is the TCF1^+^ stem-like CD4^+^ T-cell population, which emerges early after activation and serves as a reservoir for effector differentiation. These cells dynamically integrate environmental cues to direct effector differentiation and shape functional outcomes at target tissue sites, a process we define as *clonal adaptation*. By balancing self-renewal with effector differentiation, stem-like CD4^+^ T cells continue to replenish short-lived effector cells to sustain autoimmunity, transplant rejection, chronic infections, and allergic diseases. However, under tolerogenic conditions or within the tumor microenvironment, these cells often fail to differentiate into effectors, instead entering dysfunctional states or regulatory T-cell differentiation. Targeting stem-like CD4^+^ T cells offers great therapeutic potential: disrupting their persistence could mitigate autoimmune pathology and transplant rejection, whereas enhancing their effector capacity could improve antitumor immunity.

## Introduction

The adaptive immune system relies on T cells to orchestrate antigen-specific responses and maintain the balance between immunity and tolerance [1–4]. Traditional models of T-cell biology emphasize a linear progression in which antigen-driven clonal selection leads to expansion and differentiation into effector and memory populations. While these principles explain acute immune responses and memory formation, they fall short in accounting for the persistence or dysfunction of T-cell responses in chronic conditions such as autoimmunity, transplantation, allergies, chronic infections, and cancer. Additionally, these models do not fully elucidate the mechanisms underlying altered T-cell behavior in the context of peripheral immune tolerance, such as fetal-maternal tolerance, food tolerance, and recognition by the commensal microbiota. Across chronic disease states and tolerance-inducing settings, T cells exhibit distinct fates, ranging from sustained immunity to hyporesponsiveness, exhaustion, and regulatory tolerance [1–4]. This raises a fundamental question: can a unifying framework better capture the complexity of T-cell fate decisions across such diverse scenarios?

CD4^+^ T cells, with their remarkable functional diversity, are central to immune homeostasis and disease [4–6]. Unlike CD8^+^ T cells, which primarily mediate cytotoxicity, CD4^+^ T cells orchestrate immune responses through differentiation into specialized subsets, including Th1, Th2, Th17, T follicular helper (Tfh), and regulatory T (Treg) cells [4–6]. These subsets, defined by distinct transcriptional programs and cytokine profiles, have provided an overarching framework for understanding adaptive immunity. However, this classification does not fully explain the persistence or dysfunction of CD4^+^ T cells in chronic immune responses or their role in immune tolerance. For example, in tumors, CD4^+^ T cells rarely differentiate into Th1 cells and, unlike CD8^+^ T cells, do not exhibit a classical exhaustion phenotype [7, 8]. Instead, their function is often suppressed by the immunosuppressive tumor microenvironment [9, 10]. Conversely, in autoimmunity, transplantation, and allergies, CD4^+^ T-cell responses often persist despite the presence of regulatory mechanisms [11–13]. Moreover, in immune tolerance settings such as fetal-maternal tolerance and oral tolerance, CD4^+^ T cells are not merely nonresponsive but instead adopt alternative states [14, 15]. These observations highlight a critical gap in our understanding, namely, that the subset-based framework is insufficient to explain how CD4^+^ T cells behave across different immune contexts.

Recent discoveries provide a new perspective by identifying antigen-primed yet less differentiated stem-like CD4^+^ T cells, which sustain immune responses by continuously replenishing effector cells [11–13, 16]. This T-cell stemness concept, which is well established in CD8^+^ T cells, has only recently been explored in the more heterogeneous CD4^+^ T-cell compartment. Stem-like CD4^+^ T cells have been identified in various immune contexts, including transplantation, autoimmunity, allergies, cancer, and chronic infections [8, 11–13, 16, 17]. A key question emerges: How do stem-like CD4^+^ T cells integrate environmental cues and specific disease settings to fine-tune their differentiation over time? Unlike classical effector differentiation, which follows a linear trajectory toward fixed subsets, stem-like CD4^+^ T cells exhibit remarkable flexibility and adaptability in response to both acute and chronic antigen exposure, inflammatory signals, and tissue-specific cues—a process we term clonal adaptation.

While stem-like CD8^+^ T cells have been extensively reviewed elsewhere [18], this review focuses on how stem-like properties and clonal adaptation redefine CD4^+^ T-cell biology. Moving beyond traditional subset classification, we explore how these concepts explain immune plasticity, persistence, dysfunction, and therapeutic potential. By shifting from a static framework to a dynamic paradigm, we aim to identify unifying principles that govern CD4^+^ T-cell fate across diverse immune contexts. Understanding these principles has profound implications for immunotherapy, offering strategies to enhance durable responses in cancer while disrupting persistent pathogenic clones in autoimmunity and transplantation.

## Discovery and characterization of CD4^+^ T cell subsets

In the late 1970s and early 1980s, researchers identified CD4 and CD8 as key surface markers that distinguish two major populations of T cells in mice and humans [19–21]. CD4^+^ T cells recognize antigens presented by MHC class II molecules and were initially termed “helper” T cells because of their role in assisting other immune cells, particularly B cells, in effective immune responses [21–24]. In contrast, CD8^+^ T cells recognize antigens presented by MHC class I molecules and are termed cytotoxic T lymphocytes (CTLs) because of their ability to kill infected or cancerous cells directly [21, 25–27]. These early findings established the fundamental roles of CD4^+^ and CD8^+^ T cells in adaptive immunity.

**Fig. 1:**
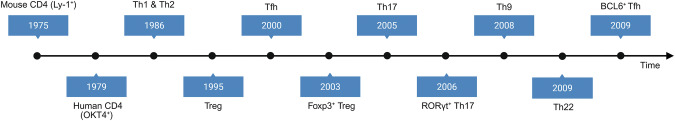
Timeline of key discoveries in CD4^+^ T-cell biology. This timeline highlights major milestones in the identification and characterization of CD4^+^ T-cell subsets. Mouse CD4^+^ T cells (Ly-1^+^, lacking Ly-2 and Ly-3) and human CD4^+^ T cells (OKT4^+^) were identified in 1975 and 1979, respectively. The Th1 and Th2 subsets were first distinguished in 1986, followed by the discovery of regulatory T (Treg) cells in 1995 and their transcriptional regulator Foxp3 in 2003. Follicular helper T (Tfh) cells were described in 2000, with BCL6 later identified as the defining transcription factor in 2009. Th17 cells were characterized in 2005, with RORγt identified as a key transcriptional regulator in 2006. Additional subsets, including Th9 and Th22 cells, were reported in 2008 and 2009, respectively. Created with BioRender.com

Subsequent studies revealed that antigen-primed CD4^+^ T cells are functionally diverse (Fig. 1). In the late 1980s, research on cytokine production led to the identification of two distinct subsets: Th1 and Th2 cells [28, 29]. This Th1–Th2 paradigm was initially described in infectious models, illustrating how the immune system tailors respond to different pathogens. Th1 cells, which are characterized by IFN-γ production, drive cell-mediated immunity by activating macrophages to eliminate intracellular pathogens such as *Mycobacterium tuberculosis* and *Leishmania major* [30, 31]. They also enhance cytotoxic responses against viral infections by promoting the activity of CD8^+^ T cells and NK cells [32, 33]. Th2 cells secrete IL-4, IL-5, and IL-13, which promote humoral immunity by inducing B-cell class switching to IgE and recruiting eosinophils to combat extracellular parasites such as helminths [34, 35].

Th1 and Th2 cells were later found to play key roles in immune-mediated diseases [36–44]. Th1 cells are major mediators of autoimmunity and alloimmune responses, contributing to the pathogenesis of multiple sclerosis, inflammatory bowel disease (IBD), rheumatoid arthritis, type 1 diabetes, graft-versus-host disease (GVHD), and organ transplant rejection [36–39]. Their ability to license dendritic cells (DCs), activate macrophages, and amplify inflammation can lead to tissue damage under these conditions. In cancer, while CD8^+^ CTLs are the primary effectors, Th1 cells enhance antigen presentation and activate cytotoxic CD8^+^ T cells, NK cells, and M1-like macrophages, fostering an effective antitumor response [40–43]. In contrast, Th2 cells play a key role in allergic diseases, including asthma and atopic dermatitis, where excessive IgE production and eosinophil activation drive chronic inflammation [44].

By the late 2000s, the discovery of Th17 and Tfh cells further expanded the diversity of CD4^+^ T-cell subsets [45–49]. Th17 cells produce IL-17A, IL-17F, IL-21, and IL-22, distinguishing them from Th1 and Th2 cells. They are essential for host defense against extracellular bacteria and fungi, particularly at mucosal surfaces, because they recruit neutrophils and enhance epithelial barrier integrity. However, their strong inflammatory properties also contribute to autoimmune and chronic inflammatory diseases. Th17 cells contribute to psoriasis pathogenesis by promoting keratinocyte hyperproliferation and the development of skin lesions. In multiple sclerosis, rheumatoid arthritis, and IBD, Th17 cells act synergistically with Th1 cells to amplify inflammation and tissue damage [45, 46].

Tfh cells specialize in providing help to B cells within germinal centers (GCs) of secondary lymphoid organs [47, 48]. They are defined by the expression of CXCR5, PD-1, ICOS, and the transcription factor Bcl-6, which enable their migration to B-cell follicles and their functional specialization. Tfh cells govern the generation of high-affinity antibodies by interacting with B cells through CD40L and ICOS and secreting IL-21 and IL-4, which are essential cytokines for B-cell proliferation, class-switch recombination, and differentiation into plasma cells. These processes are critical for effective humoral immunity and vaccine responses. However, dysregulated Tfh cell activity is linked to autoimmune diseases such as systemic lupus erythematosus (SLE) and rheumatoid arthritis, where excessive B-cell activation leads to the production of pathogenic autoantibodies [47, 48, 50]. The identification of Th17 and Tfh cells challenges the “binary” Th1/Th2 model, underscoring the specialized roles of CD4^+^ T-cell subsets in adaptive immunity.

The discovery of Treg cells marked another milestone in CD4^+^ T-cell research, providing critical insights into immune regulation. In 1995, Treg cells were identified as a subset of CD4^+^ T cells expressing high levels of CD25, and in 2003, Foxp3 was established as a master regulator that is essential for Treg development and function [51–54]. Treg cells are generated primarily in the thymus but can also be induced in the periphery under specific tolerogenic conditions. Their main function is to maintain immune tolerance and limit inflammation. Unlike effector T cells, which drive immune responses, Treg cells act as a counterbalance, ensuring immune homeostasis and preventing immunopathology [55]. Their discovery further expands the functional diversity of CD4^+^ T cells and the intricate regulatory mechanisms that keep immune responses in check.

**Fig. 2:**
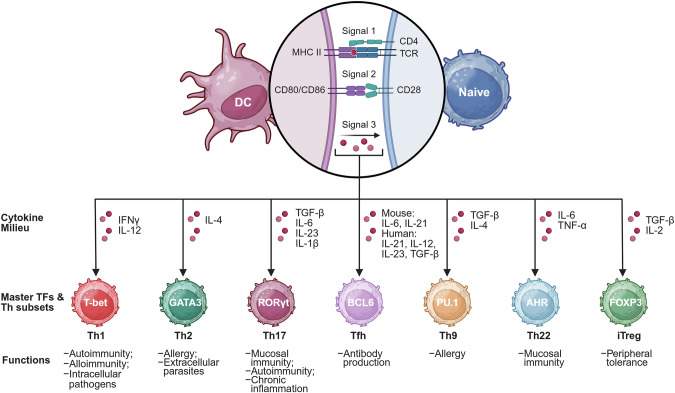
Differentiation of naïve CD4^+^ T cells into distinct effector subsets. Upon encountering antigen-presenting dendritic cells (DCs), naïve CD4^+^ T cells receive three key signals that drive their activation and differentiation. Signal 1 involves recognition of peptide‒MHC class II complexes by the T-cell receptor (TCR), whereas signal 2 is delivered through costimulatory molecules such as CD28 engaging with CD80/CD86. Signal 3 consists of cytokines in the surrounding microenvironment, which direct lineage-specific differentiation. Distinct cytokines induce the expression of master transcription factors, thereby driving the development of specific CD4^+^ T helper (Th) cell subsets, including Th1, Th2, Th17, Tfh, Th9, and Th22, and induced regulatory T (iTreg) cells. The representative transcription factors, polarizing cytokines, and immune functions associated with each subset are indicated. Adapted from the ‘T-cell activation and differentiation’ template on BioRender.com (2020)

The landscape of CD4^+^ T-cell subsets continues to expand (Fig. 2). Th9 cells, defined by their production of IL-9, play roles in antiparasitic immunity and are implicated in allergic inflammation and tumor immunity [56]. Th22 cells, which are characterized by their production of IL-22, contribute to skin barrier function and have been linked to psoriasis and other inflammatory skin diseases [57]. Additionally, cytotoxic CD4^+^ T cells, which express granzymes and perforin, have been identified as a subset capable of directly killing target cells, expanding the functional roles of CD4^+^ T cells beyond classical helper functions [58].

While the classification of CD4^+^ T cells into diverse subsets illustrates their functional flexibility in response to different immune challenges, this subset-based framework falls short of explaining how CD4^+^ T cells maintain long-term responses, shift between functional states, or contribute to immune dysfunction and tolerance.

## The evolving concept of T cell stemness

T-cell stemness refers to the ability of certain T cells to exhibit stem cell-like properties, including self-renewal, multipotency, and long-term persistence. This concept has emerged to explain mechanisms that sustain durable immune responses, particularly in the context of memory formation, chronic infection, and cancer immunotherapy. While naïve T cells serve as the “ancestors” of all effector and memory subsets, T-cell stemness pertains to antigen-experienced T cells that retain the capacity to self-renew and continuously generate both effector and memory progenies. Genes such as *Tcf7*, *Lef1*, *Id3*, *Klf2*, *Il7r*, *Sell*, *Ccr7*, and *Slamf6* are associated with T-cell stemness, most of which are inherited from naïve T cells, whereas a few are de novo expressed upon T-cell activation [11].

As early as 2001, Fearon et al. proposed that a subset of antigen-experienced lymphocytes retains self-renewal through “arrested differentiation”, allowing them to sustain long-term immune responses [59]. This principle is exemplified in the memory T-cell compartment, where central memory T (T_CM_) cells retain self-renewal capacity, whereas effector memory T (T_EM_) cells are more committed to differentiation. Identifying transcriptional regulators that govern arrested differentiation in T cells could provide a molecular framework for T-cell stemness, offering new strategies to increase immune persistence in chronic infections and enhance tumor immunity [59].

The stem cell-like properties of CD8^+^ T_CM_ cells have been demonstrated through serial single-cell adoptive transfer and infection-driven re-expansion experiments, confirming their ability to self-renew and generate diverse progeny, including both effector and memory cells. Compared with T_EM_ cells, T_CM_ cells exhibit superior proliferative and differentiation potential, underscoring their critical role in sustaining adaptive immunity [60]. By expanding this framework, researchers have identified memory stem T (T_SCM_) cells, a population that is even less differentiated than T_CM_ cells [61–63]. As the least differentiated subset within the memory T-cell hierarchy, T_SCM_ cells exhibit enhanced self-renewal and multipotency, resembling features of naïve T cells despite being antigen-experienced. Unlike more differentiated memory subsets, T_SCM_ cells maintain long-term persistence and can generate all memory and effector T-cell subsets. Their superior capacity for immune reconstitution has been demonstrated in hematopoietic stem cell transplantation and adoptive immunotherapy, where they contribute to the long-term persistence of transferred T cells [62, 63].

The concept of T-cell stemness has further evolved in the context of chronic antigen exposure, particularly through studies of chronic lymphocytic choriomeningitis virus (LCMV) infection. For over three decades, the chronic LCMV model has been instrumental in defining T-cell exhaustion, a state of progressive dysfunction caused by protracted antigen stimulation [64, 65]. However, this raises a fundamental question: how can stemness, which implies self-renewal and multipotency, coexist with exhaustion, a state characterized by diminished function?

A key breakthrough occurred in 2016 when three seminal studies identified a subset of virus-specific CD8^+^ T cells that express TCF1 and function as progenitors during chronic LCMV infection [66–68]. These TCF1^+^ cells reside primarily in lymphoid tissues, where the niche supports their self-renewal and ongoing differentiation. Unlike fully exhausted T cells, which lose their proliferative capacity, TCF1^+^ progenitors retain the ability to both expand and differentiate. These cells sustain chronic immune responses and serve as the primary cell population that responds to PD-1 blockade immunotherapy [66, 67].

The identification of TCF1^+^ progenitors reshaped the understanding of T-cell stemness and exhaustion during chronic antigen exposure [18]. Rather than an abrupt loss of function, T-cell exhaustion represents a gradual shift in differentiation. Initially, stem-like TCF1^+^ progenitors generate functional effector cells, but over time, their differentiation trajectory skews toward the production of increasingly exhausted progeny. This realization has prompted efforts to manipulate TCF1^+^ progenitors to enhance immune responses in chronic infections and cancer. In addition to PD-1 blockade, IL-2-related therapies and other interventions are being explored to promote their ability to promote effector differentiation rather than exhaustion [69, 70].

Given the central role of TCF1^+^ progenitors, it is important to clarify the terminology used to describe them. During chronic antigen exposure, these cells are termed memory-like T cells, progenitors of exhausted T cells, precursors of exhausted T cells, or stem-like T cells [18, 71–73]. Interestingly, similar TCF1^+^ cells also emerge early during acute LCMV infection, exhibiting nearly identical characteristics to their counterparts in chronic infections [74, 75]. However, in acute infection settings, these cells primarily differentiate into effector T cells rather than progressing toward exhaustion. Owing to their conserved role in sustaining immune responses and driving effector differentiation, we refer to these cells as “stem-like” T cells throughout this review, regardless of whether antigen exposure is acute or chronic. Unlike conventional memory T cells, which arise after antigen clearance, stem-like T cells emerge early upon antigen stimulation and serve as a progenitor pool that gives rise to effector, memory, and exhausted T-cell populations, depending on the context in which the T cells are stimulated [18, 71–76].

Consistent with Fearon et al.’s theory [59], TCF1 is the key transcription factor that maintains stem-like CD8^+^ T-cell identity by enforcing differentiation arrest, although the underlying molecular mechanisms remain to be fully elucidated [77, 78]. However, TCF1 is not the only transcriptional regulator involved in this process. For example, c-Myb activates *Tcf7* (encoding TCF1) to increase stemness while repressing *Zeb2* to restrain effector differentiation [79]. BACH2 reinforces the stem-like program by binding to enhancers of TCR-driven genes, thereby limiting AP-1 accessibility to Jun family transcription factors and restraining effector differentiation [80]. Additional factors, such as BCL6 and Id3, likely contribute to differentiation arrest as well [68, 81].

Overall, the concept of stem-like T cells has been extensively studied in the context of CD8^+^ T cells. However, its relevance to CD4^+^ T cells is only beginning to emerge and will be discussed in the following sections.

## Stem-like CD4^+^ T cells and their relationship to helper subsets

### Th1 responses in infection

Before the term “stem-like T cells” was widely adopted and TCF1 was recognized as a key marker, a seminal 2015 study identified a memory-like CD4^+^ T-cell population during *Mycobacterium tuberculosis* (Mtb) infection [82]. In some cases, tuberculosis immunology has focused primarily on enhancing Th1 responses, as IFN-γ-producing CD4^+^ T cells are considered critical for protection. However, the failure of vaccines such as MVA85A, which boosts Th1 responses but does not improve protection, highlights the need to reevaluate which CD4^+^ T-cell subsets sustain long-term immunity. Using MHC-II tetramers, two distinct populations of Mtb-specific CD4^+^ T cells that persist under chronic antigen stimulation were identified: KLRG1^+^ terminally differentiated Th1 cells and PD-1^+^KLRG1^‒^ memory-like cells. Adoptive transfer experiments revealed striking functional differences between these subsets. KLRG1^+^ cells exhibited limited proliferation and primarily generated KLRG1^+^ progeny. In contrast, PD-1^+^KLRG1^‒^ cells proliferated extensively, self-renewed, and gave rise to KLRG1^+^ effectors, providing superior protection against Mtb infection compared with KLRG1^+^ cells. PD-1^+^KLRG1^‒^ cells also share molecular features with Tfh cells, expressing ICOS, Bcl6, and CXCR5, all of which contribute to their persistence during chronic infection [82].

**Fig. 3:**
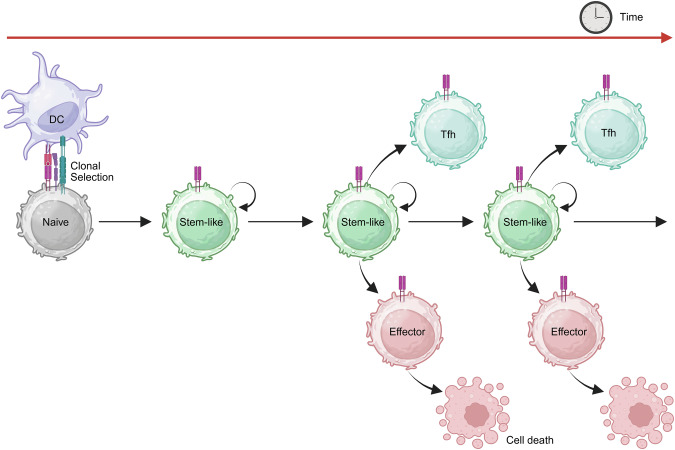
Stem-like CD4^+^ T cells serve as a reservoir for sustained effector differentiation. Following clonal selection by dendritic cells (DCs), naïve CD4^+^ T cells differentiate into a stem-like population that functions as a self-renewing reservoir. These stem-like cells maintain themselves while progressively giving rise to functionally distinct progeny. Over time, they differentiate into either terminal effector Th cells (e.g., Th1), which execute immune functions and ultimately undergo cell death, or follicular helper T (Tfh) cells, which support B-cell responses. This model highlights the central role of the stem-like CD4^+^ T-cell state in sustaining adaptive immunity through self-renewal and continuous output of effector Th and Tfh lineages. Created with BioRender.com

The identification of TCF1 as a key marker advanced the study of stem-like CD4^+^ T cells, refining our understanding of their role in sustaining Th1 responses. In the context of infection, an early study examined how TCF1^+^ CD4^+^ T cells function as Th1 progenitors during influenza infection [83]. Using an OT-II TCR transgenic system and PR8-OVA influenza virus, the authors showed that TCF1 expression persists through early divisions of antigen-specific CD4^+^ T cells in the draining lymph nodes (DLNs). After four to five divisions, cells bifurcate into TCF1^+^ and TCF1^‒^ populations, with TCF1^+^ cells retaining self-renewal capacity and TCF1^‒^ cells committing to Th1 effector differentiation and lung infiltration. While TCF1^+^ cells generate both progenitor-like and effector progeny, TCF1^‒^ cells cannot revert to a progenitor state [83]. Therefore, naïve CD4^+^ T cells give rise to TCF1^+^ stem-like cells with self-renewal and effector differentiation capacities (Fig. 3).

In a chronic LCMV infection model, a recent study revealed a population of PD-1^+^TCF1^+^ CD4^+^ T cells with stem-like properties [16]. TCR clonal tracing and adoptive transfer experiments demonstrated that these cells possess self-renewal capacity and function as progenitors, continuously generating Teff and Tfh cells during chronic antigen stimulation. Conditional deletion of *Bcl6* revealed that the development of these genes depends on BCL6. Although these studies broadly refer to their effector progeny as Teffs, these Teffs express T-bet and CXCR6, which are markers indicative of Th1-like functionality [16]. Notably, the authors suggested that naïve CD4^+^ T cells can directly differentiate into TCF1^‒^ early effector cells [16], similar to what has been reported for CD8^+^ T cells [84].

In contrast, in the influenza infection model, antigen-specific CD4^+^ T cells bifurcate into TCF1^+^ and TCF1^–^ populations only after four to five divisions [83]. Furthermore, in tumor and organ transplantation models, TCF1^–^ cells emerge from TCF1^+^ stem-like cells only after extensive proliferation, possibly due to the absence of strong infection-induced stimuli [11, 16]. Therefore, whether naïve CD4^+^ T cells can directly differentiate into early effector cells remains an open question.

### Th1 responses in autoimmunity

In 2018, an elegant study using IFNγ-Thy1.1 reporter mice revealed a stem-like subset of CD4^+^ T cells responsible for initiating and maintaining IBD [85]. In this study, CD4^+^ T cells were isolated from mice with established IBD and transferred into new hosts. Strikingly, the transfer of IFNγ-nonproducing (Thy1.1^‒^) cells, not IFNγ-producing (Thy1.1^+^) cells, was capable of conferring pathogenicity. These pathogenic Thy1.1^‒^ cells exhibited a transcriptional signature of stemness, reflecting their enhanced self-renewal and survival. Importantly, they continuously generate terminally differentiated IFN-γ-producing Th1 cells within the inflamed intestine [85].

A recent study investigated how autoimmune CD4^+^ T cells drive type 1 diabetes (T1D) progression in NOD mice [86], a disease dominated by Th1 responses. Unlike infection-driven responses, autoimmune CD4^+^ T cells retain TCF1 expression during antigen priming, likely due to the absence of inflammatory signals associated with infections. This study further revealed that autoimmune effector responses in pancreatic tissues are sustained by a continuous influx of peripheral stem-like CD4^+^ T cells, which replenish the infiltrate and maintain its functional potential after entering the tissue. A key focus is how TCF1 expression is fine-tuned to balance persistence and effector function. While islet-infiltrating stem-like CD4^+^ T cells retain TCF1 expression, their TCF1 expression is lower than that of naïve T cells. This gradual decline is preprogrammed by partial de novo methylation of the *Tcf7* locus in circulating autoimmune CD4^+^ T cells. By maintaining reduced TCF1 expression, islet-infiltrating stem-like CD4^+^ T cells balance self-renewal with effector differentiation, ensuring long-term persistence despite chronic antigen exposure [86].

In human ulcerative colitis (UC), TCF1^+^ stem-like CD4^+^ T cells are directly implicated in disease pathogenesis [12]. Colonic T cells from UC patients exhibit gene expression profiles similar to those of murine stem-like progenitors. These stem-like T cells are enriched in inflamed regions of the colon, and TCR sequencing revealed that they are clonally related to proinflammatory effector T cells, suggesting that they serve as a reservoir for sustained inflammation. Using a murine adoptive transfer-induced colitis model, the authors further demonstrated that CD4^+^ T cells lacking TCF1 or BCL6 presented reduced colonic infiltration and diminished pathogenicity. Thus, TCF1 and BCL6 are critical for sustaining CD4^+^ T-cell stemness and maintaining chronic inflammation in UC [12].

In human autoimmune vasculitis, TCF1^+^ stem-like CD4^+^ T cells have been identified within tertiary lymphoid structures surrounding the adventitial vasa vasora [87]. These cells exhibit high proliferative capacity and give rise to two key effector populations: EOMES^+^ cytotoxic T cells and BCL6^+^ Tfh-like cells. The use of IL-7R as a surrogate marker for TCF1^hi^ CD4^+^ T cells revealed that these stem-like CD4^+^ T cells are essential for driving transferable and persistent vasculitis in serial transplantation experiments. These findings establish TCF1^+^ stem-like CD4^+^ T cells as key drivers of disease chronicity in autoimmune vasculitis [87].

**Fig. 4:**
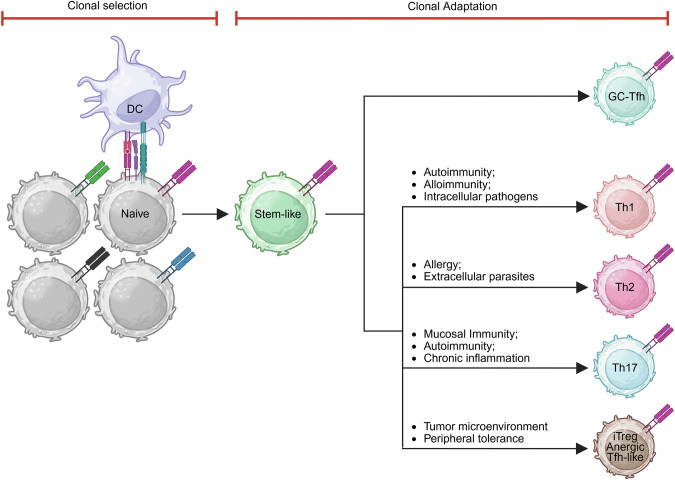
Clonal selection and adaptation drive the diversification of CD4^+^ T-cell fates. Naïve CD4^+^ T cells expressing diverse T-cell receptors (TCRs) undergo clonal selection upon recognition of cognate antigens presented by dendritic cells (DCs). This initial selection leads to the generation of a stem-like CD4^+^ T-cell population, which serves as a self-renewing reservoir. These stem-like cells differentiate into specialized effector subsets in response to environmental cues—a process termed **clonal adaptation** in this review. These subsets include germinal center T follicular helper (GC-Tfh) cells that support B-cell responses; Th1 cells involved in autoimmunity, alloreactivity, and responses to intracellular pathogens; Th2 cells that mediate allergy and defense against extracellular parasites; Th17 cells that function in mucosal immunity, autoimmunity, and chronic inflammation; and regulatory or dysfunctional states, such as induced regulatory T (iTreg), anergic, or Tfh-like cells found in tolerogenic or tumor microenvironments. Created with BioRender.com

Collectively, these studies highlight the essential role of stem-like CD4^+^ T cells in sustaining Th1 responses in autoimmunity. These cells are capable of self-renewal while continuously generating Tfh and effector Th1 cells (Fig. 4). Understanding their regulatory mechanisms will be crucial for developing targeted therapies to modulate immune responses in these diseases.

### Th1 responses in alloimmunity

In allogeneic hematopoietic stem cell transplantation (HSCT) and solid organ transplantation, T cells are continuously exposed to alloantigens, often throughout the recipient’s lifetime. Unlike chronic infections or cancer, where persistent antigen stimulation drives T-cell exhaustion, alloantigen-specific T cells in transplantation remain functional over time. The mechanisms that allow these cells to avoid exhaustion under sustained alloantigen exposure are only beginning to be understood [11, 88–90].

One study investigated how donor CD4^+^ T cells differentiate and function during acute gastrointestinal GVHD after allogeneic HSCT in a mouse model [89]. Using single-cell RNA sequencing, this study identified two primary differentiation trajectories among alloreactive CD4^+^ T cells in mesenteric lymph nodes (mLNs): one leading to proinflammatory and regulatory effector states expressing IFN-γ, IL-17A, or Foxp3 and the other forming a quiescent, TCF1^hi^ population with reduced cytokine expression and metabolic activity. These TCF1^hi^ cells expressed stemness-associated genes, retained the potential to differentiate into effector cells upon secondary stimulation, and migrated to the gut while maintaining their transcriptional profile [89]. These findings suggest that alloimmune CD4^+^ T cells exhibit heterogeneous differentiation fates, with the TCF1^hi^ state potentially playing a critical role in long-term persistence and function during GVHD.

Another study investigated how GVHD is sustained at targeted tissue sites [90]. Using T-cell clone tracking, parabiosis experiments, and computational modeling in a mouse model, researchers have shown that GVHD persists primarily through a small subset of tissue-resident TCF1^+^ progenitor-like T cells rather than through continuous recruitment of alloreactive T cells from the circulation. These progenitor-like cells self-renew and differentiate into effectors, fueling the local inflammatory response. By demonstrating how GVHD endures despite chronic antigen exposure, this study challenges the prevailing model of GVHD maintenance and suggests new therapeutic strategies targeting progenitor T cells in local tissues [90].

Two distinct studies provided insights into the transcriptional regulation of stem-like CD4^+^ T cells in GVHD. The first study highlights the essential role of TCF1 in maintaining the stemness and functional persistence of CD4^+^ T cells. Deletion of TCF1 in these cells impairs their ability to drive GVHD and cause damage to target organs [91]. The second study revealed that Id3 is essential for maintaining TCF1^+^ stem-like CD4^+^ T cells in GVHD target tissues. Deletion of Id3 leads to increased PD-1 expression in Th1 cells, promoting their dysfunction, and reduces the number of tissue-infiltrating TCF1^+^ stem-like cells, ultimately inhibiting GVHD progression. Mechanistically, Id3 maintains stemness by reducing the chromatin accessibility of key transcription factors that promote PD-1 transcription, effector differentiation, and dysfunction. Thus, Id3 plays a critical role in balancing self-renewal, differentiation, and dysfunction in stem-like CD4^+^ T cells during GVHD [92].

The stem-like properties of CD4^+^ T cells can be conceptualized through two distinct programs: one that maintains their identity and self-renewal capacity and the other that governs their differentiation into effector cells. It remains unclear whether these programs coexist within individual stem-like CD4^+^ T cells and when they begin to diverge, potentially through mechanisms such as asymmetric cell division. Despite these uncertainties, the molecular basis of these programs has recently been elucidated in the context of organ transplantation, where CD4^+^ T cells play a critical role in mediating allograft rejection [11].

In a heart transplantation model, single-cell transcriptomic analysis revealed that naïve alloantigen-specific CD4^+^ T cells (TCR transgenic TEa cells) differentiate into two major populations: TCF1^+^Ly108^+^ effector precursor T (T_EP_) cells and TCF1^‒^CXCR6^+^ effector cells [11]. Tracing cell proliferation shows that only cells that have undergone extensive division (indicated by the loss of tracing dye) can differentiate into effector cells. Once differentiated, effector cells exhibit all the cardinal features of effector activities but lose their proliferative capacity and become prone to apoptosis, preventing them from sustaining alloreactivity or mediating transplant rejection in adoptive transfer models. In contrast, T_EP_ cells exhibit continuous self-renewal and generate large numbers of effector cells, enabling them to drive rejection even after sequential transfers into secondary and tertiary hosts [11]. Thus, stem-like T_EP_ cells serve as a persistent source of effector cells, making transplant rejection critically dependent on their ability to replenish the effector pool (Fig. 3).

TCF1 preserves the identity and self-renewal capacity of stem-like T_EP_ cells [11]. Since TCF1 prevents T_EP_ cells from differentiating into effector cells, its silencing via H3K27me3 is required for effector differentiation. Surprisingly, the transcription factor IRF4 is involved in this silencing process; in its absence, virtually alloreactive T cells remain arrested in the TCF1^+^ T_EP_ cell state. Additionally, the metabolic enzyme LDHA is essential for enabling T_EP_ cells to differentiate into effectors. Ablation of either IRF4 or LDHA in T cells prevents effector generation and leads to transplant tolerance [11]. Together, these findings reveal that the identity/self-renewal and effector potential of stem-like T cells are regulated by distinct molecular programs.

How TCF1^‒^CXCR6^+^ effector cells mediate graft destruction remains unclear. While these cells produce IFN-γ, their differentiation occurs independently of T-bet, suggesting a noncanonical Th1-like differentiation pathway [11]. Notably, TCF1^‒^CXCR6^+^ effector cells have also been identified across various CD4^+^ T-cell-mediated immune responses, highlighting the need for further studies to define their molecular regulation and precise role in driving tissue damage in autoimmunity and allograft rejection [11, 93, 94].

### Th2 responses

Over the past year, three studies have introduced the concept of stem-like T cells into the Th2 research field (Fig. 4) [13, 95, 96]. One study tracked CX3CR1^+^CD4^+^ T cells in helminth infection models, identifying them as an activated, tissue-homing population with diverse cytokine production capacities. Single-cell RNA sequencing revealed heterogeneity within this population, including a distinct BCL6^+^TCF1^+^PD1^+^ subset in the spleen. Notably, while CX3CR1 is typically associated with terminally differentiated T cells, the BCL6^+^TCF1^+^ subset within the CX3CR1^+^CD4^+^ population exhibited progenitor-like features, suggesting that not all CX3CR1^+^CD4^+^ T cells are terminal effectors. Targeted deletion of BCL6 within this subset reduces the frequency of CX3CR1^+^CD4^+^ T cells during infection, underscoring the role of BCL6 in sustaining Th2 responses to helminth infections [95].

Another study identified a multipotent progenitor population of Th2 cells, termed Th2-multipotent progenitors (Th2-MPPs), which coexpress TCF1 and LEF1 and are present in multiple human allergic diseases [13]. These Th2-MPP cells exhibit self-renewal capacity and the ability to differentiate into Th2 effector, Tfh, and Treg cells. Single-cell TCR lineage tracing further revealed clonal relationships between Th2-MPPs and differentiated subsets, supporting their progenitor-like identity. Interestingly, Th2-MPP cells are resistant to IL-4 receptor blockade but expand in response to thymic stromal lymphopoietin (TSLP), which also protects them from glucocorticoid-induced apoptosis [13]. These findings position Th2-MPP cells as critical drivers of chronic type 2 inflammation, suggesting that persistent Th2 responses in allergic diseases may be maintained by a stem-like cell population rather than terminally differentiated effector cells.

A recent study identified hypoxia-inducible factor 2α (HIF2α) as a key regulator of pathogenic Th2 cell differentiation and function [96]. In patients with asthma and chronic rhinosinusitis, HIF2α is highly expressed in Th2 cells, suggesting its involvement in disease pathology. Using mouse models, the study demonstrated that HIF2α is essential for the transition of TCF1^+^Ly108^+^ stem-like cells into pathogenic Th2 cells. Deletion of HIF2α impaired this transition, reduced airway inflammation, and decreased the accumulation of pathogenic Th2 cells. Mechanistically, HIF2α collaborates with GATA3 to regulate phospholipid metabolism and enhance TCR-PI3K-AKT signaling by promoting the transcription of inositol polyphosphate multikinase (IPMK). The overexpression of IPMK in HIF2α-deficient cells restored pathogenic Th2 differentiation, whereas the pharmacological inhibition of HIF2α suppressed airway inflammation [96].

Collectively, these studies highlight the critical role of stem-like CD4^+^ T-cell populations in sustaining Th2 responses across allergies, parasite infections, and chronic inflammatory diseases. They reveal diverse mechanisms, including transcriptional regulation by factors such as BCL6 and HIF2α, as well as cytokine signaling pathways such as TSLP, that maintain the self-renewal and differentiation potential of these cells [13, 95, 96]. These findings provide new insights into the persistence of type 2 inflammation and identify potential therapeutic targets for intervention.

### Stem-like Th17 cells

As early as 2011, two studies challenged the conventional view that Th17 cells are short-lived and terminally differentiated [97, 98]. One study demonstrated that Th17 cells possess stem cell-like properties, persisting long term with a molecular signature resembling that of early memory CD8^+^ T cells. These cells express high levels of *Tcf7* and β-catenin, undergo self-renewal, and differentiate into Th1-like effector progeny, processes that are essential for tumor eradication and autoimmune responses [97]. Another study revealed that human Th17 cells persist in chronic disease settings, such as GVHD, ulcerative colitis, and cancer. These cells exhibit proliferative self-renewal, resistance to apoptosis, and the ability to differentiate into other Th subsets. Mechanistically, HIF-1α and Notch have been identified as key regulators of antiapoptotic gene expression in human Th17 cells, further supporting their long-lived and adaptable nature [98].

A recent study explored the metabolic heterogeneity of Th17 cells and its impact on autoimmune diseases [99]. Using a mouse model of experimental autoimmune encephalomyelitis (EAE), the authors identified two distinct Th17 subsets: a stem-like CD27^+^ TCF1^hi^ subset with lower anabolic metabolism and a CD27^‒^ T-bet^hi^ subset with higher metabolic activity that promotes transdifferentiation into Th1-like cells. Th17 cells with disrupted mTOR1 signaling or impaired anabolic metabolism are arrested in the TCF1^+^ stem-like state, failing to develop into Th1-like cells and losing their ability to mediate autoimmune neuroinflammation [99]. These findings emphasize the crucial role of metabolism in balancing Th17 stemness and effector differentiation, shaping their contribution to autoimmune pathogenesis.

Another study investigated the heterogeneity, functional plasticity, and migratory behavior of intestinal Th17 cells in autoimmune neuroinflammation [93]. Using single-cell RNA and TCR sequencing combined with fate mapping, the authors identify a stem-like, homeostatic population of TCF1^+^Ly108^+^ Th17 cells maintained by the microbiota in the intestine. These cells serve as a reservoir for the generation of pathogenic GM-CSF^+^ IFNγ^+^ CXCR6^+^ Th17 cells, which migrate to the central nervous system (CNS) and drive EAE. IL-23 signaling is required for the differentiation of these stem-like Th17 cells into encephalitogenic effector cells [93]. This study establishes a direct in vivo lineage relationship between homeostatic stem-like Th17 cells and their pathogenic progeny, providing mechanistic insight into how microbiota-maintained intestinal Th17 cells contribute to extraintestinal autoimmune disease.

To further elucidate Th17 regulation, a recent study focused on the transcription factor TCF1 and its role in maintaining Th17 homeostasis [100]. The authors demonstrated that TCF1 is highly expressed in homeostatic Th17 cells and is essential for maintaining their nonpathogenic state. IL-23 signaling suppresses TCF1 expression, promoting its transition to a proinflammatory Th17 state. Mechanistically, TCF1 binds to and inhibits RORγt in a DNA-dependent manner, thereby limiting its ability to mediate inflammatory gene expression. Using a conditional deletion model (*Tcf7* knockout in mature T cells), the authors show that loss of TCF1 increases Th17 pathogenicity, even in the absence of IL-23 signaling, and exacerbates EAE when TCF1-deficient Th17 cells are transferred into recipient mice. Conversely, sustained TCF1 expression reduces encephalitogenic potential, suppressing CNS inflammation [100]. These findings establish TCF1 as a critical regulator of Th17 cell fate, orchestrating a transcriptional network that balances tissue homeostasis and inflammation.

Collectively, Th17 cells possess stem-like properties, allowing them to persist and differentiate into other Th subsets in response to changing environmental and metabolic cues.

### Tfh cells

Tfh cells are commonly defined as CXCR5^+^Bcl6^+^ CD4^+^ T cells, regardless of their localization [48]. A subset of these cells, known as GC-Tfh cells, reside within GCs and are characterized by increased expression of Bcl6 and PD-1 (CXCR5^+^Bcl6^+^PD-1^hi^). The central role of GC-Tfh cells is to provide essential signals and cytokines, such as CD40L and IL-21, to B cells within GCs, promoting their survival, affinity maturation, class-switch recombination, and selection into long-lived plasma cells and memory B cells [48]. Pre-Tfh cells, which are found in T-cell zones or interfollicular regions, represent precursor populations that have not yet entered GCs but may differentiate into GC-Tfh cells. In human studies, circulating Tfh cells, a subset of CXCR5^+^Bcl6^+^ CD4^+^ T cells in the blood, serve as biomarkers for immune responses, including vaccine efficacy, autoimmunity, and infections [101].

Within GCs, Tfh cells are thought to be largely quiescent. However, a recent study demonstrated that GC-Tfh cells undergo antigen-dependent selection, leading to dynamic proliferative expansion and contraction throughout the GC reaction [102]. This finding does not contradict the observation that fully committed GC-Tfh cells (e.g., the CXCR5^+^Bcl6^+^PD-1^hi^ subset) lose their ability to proliferate [103]. Rather, it highlights the heterogeneity and dynamic nature of Tfh cells within GCs, suggesting that their behavior is more complex than previously thought.

The exact relationship between Tfh cells and other Th subsets remains a topic of debate [48]. Emerging evidence suggests that stem-like CD4^+^ T cells possess bifurcating potential and can differentiate into either Tfh cells or Th1/Th2 cells [13, 16]. However, two critical questions remain unresolved: (1) at what stage does this bifurcation occur, and (2) can committed Tfh cells later transition into other Th subsets?

A recent study classified CXCR5^+^Bcl6^+^ Tfh cells into CD62L^+^PD-1^lo^ memory-like and PD-1^hi^ effector subsets [104]. CD62L^+^ Tfh cells emerge early during the immune response and gradually become the predominant Tfh population in the memory phase. These cells express stemness-associated genes and can readily generate PD-1^hi^ effector Tfh cells during recall responses. These findings suggest that CD62L^+^ Tfh cells occupy a hierarchical position as a precursor-like population within the Tfh lineage [104]. If so, they may represent a transitional stage in which Tfh cells begin to branch out from a broader pool of stem-like CD4^+^ T cells.

Another study explored the fates of Tfh cells under B-cell-deficient conditions and demonstrated that Tfh cells regulate colitis development by interacting with DCs in colonic lymphoid follicles [94]. Tfh cells promote the accumulation of mature DCs, whereas DCs support Tfh cell differentiation, establishing a reciprocal regulatory loop. Notably, Tfh cells can differentiate into long-lived pathogenic Th1 cells upon migrating to the lamina propria, highlighting that Tfh cells may play broader immunoregulatory roles beyond B-cell help [94].

Collectively, Tfh cells exhibit plasticity, with their fate further influenced by their location, whether within GCs or in non-GC environments. However, the developmental and functional relationships between GC-Tfh and Tfh cells in non-GC environments remain unclear.

## Beyond helper subsets: context-driven fates of stem-like CD4^+^ T cells

### Tumor control and immune evasion

CD4^+^ T cells orchestrate cancer immunity by enhancing antigen presentation, supporting CD8^+^ T-cell responses, activating innate immune cells, and directly killing tumor cells via granzyme and perforin [10, 58]. However, their function is often limited by suboptimal activation and poor tumor infiltration. The tumor microenvironment (TME) further suppresses their activity through immunosuppressive cytokines, metabolic stress, and inhibitory checkpoints, promoting their differentiation into regulatory or dysfunctional states (Fig. 4) [10, 105, 106]. Given that stem-like CD4^+^ T cells give rise to diverse effector and dysfunctional populations, we focus on their differentiation fates to understand the mechanisms underlying tumor control and immune evasion.

TCR-transgenic Trp1 CD4^+^ T cells recognize the melanoma antigen tyrosinase-related protein 1 (Trp1). Adoptive transfer of 50,000 naïve Trp1 T cells into *Rag1*^‒/‒^ mice effectively eradicates established B16 melanomas [17]. In this potent tumor control model, naïve Trp1 T cells first differentiate into TCF1^+^Ly108^+^ stem-like cells, which subsequently give rise to TCF1^–^CXCR6^+^ Th1-like effector cells. This effector differentiation is required for tumor clearance; deletion of LDHA, an enzyme that governs aerobic glycolysis, impairs effector generation and abolishes antitumor activity. However, these effector cells are short-lived, and the adoptive transfer of effector cells alone into new hosts fails to eliminate tumors. Instead, durable tumor control requires continuous replenishment of the effector pool by the stem-like population [17]. The precise mechanisms by which effector CD4^+^ T cells mediate tumor rejection remain incompletely defined. Trp1 T cells may act through both direct cytotoxicity in an MHC class II-dependent manner and indirect mechanisms such as myeloid cell reprogramming, highlighting the functional versatility of CD4^+^ T cells in antitumor immunity [107, 108].

Unlike highly functional Trp1 T cells, CD4^+^ T cells in most other tumor models exhibit hyporesponsiveness, largely due to the immunosuppressive TME. A recent study revealed TCF1^+^ stem-like CD4^+^ T cells in human kidney, bladder, and prostate tumors, highlighting their potential role in tumor immunity [8]. To investigate the fate of these cells, the authors used the LCMV glycoprotein (GP) as a model antigen in murine models. They engineered TRAMPC1 prostate and B16F10 melanoma cells to express GPs and employed the I-A^b^GP_66_ tetramer and SMARTA transgenic system to track antigen-specific CD4^+^ T-cell responses. Intriguingly, Treg cells actively suppress the differentiation of stem-like CD4^+^ T cells into Th1 effector cells, instead promoting their conversion into induced Treg (iTreg) cells over time. Upon Treg depletion, stem-like CD4^+^ T cells differentiate more robustly into Th1 cells, which in turn enhances the effector differentiation of stem-like CD8^+^ T cells in tdLNs [8]. These findings demonstrate that local Treg-mediated suppression of stem-like CD4^+^ T-cell effector differentiation contributes to immune evasion and that relieving this suppression enhances antitumor immunity.

Immune checkpoint blockade (ICB) therapies have transformed clinical cancer treatment and significantly improved outcomes for patients with advanced-stage disease. However, many patients still fail to achieve lasting clinical benefit [109]. Understanding how CD4^+^ T cells respond to cancer immunotherapies is crucial for optimizing treatment strategies and promoting durable antitumor immunity.

In addition to reinvigorating stem-like CD8^+^ T_PEX_ cells, recent studies have highlighted important roles for CD4^+^ T cells in mediating responses to PD-1/PD-L1 blockade [110, 111]. In hepatocellular carcinoma, CXCL13^+^ CD4^+^ Th cells are enriched in the tumors of patients who respond to anti-PD-1 therapy, where they support CD8^+^ T-cell expansion and effector differentiation [110]. In another study of bladder cancer patients treated with anti-PD-L1 therapy, the clinical response was instead associated with the presence of cytotoxic CD4^+^ T cells, rather than CD8^+^ T cells, within tumors [111]. These cytotoxic CD4^+^ T cells are clonally expanded, exhibit MHC class II-restricted tumor-killing activity, and are correlated with improved therapeutic outcomes [111].

T cells respond differently to distinct immunotherapies. In human melanoma, PD-1 blockade predominantly expands exhausted-like CD8^+^ T cells, increasing their proliferation within tumors. In contrast, CTLA-4 blockade induces the expansion of ICOS^+^ Th1-like CD4^+^ effector cells while also targeting specific subsets of exhausted-like CD8^+^ T cells [112]. These findings demonstrate that anti-CTLA-4 and anti-PD-1 therapies engage distinct immune cell populations, supporting the rationale for combination strategies to maximize antitumor immunity. However, dual ICB is often associated with increased immune-related adverse events [113], necessitating a careful balance between enhanced therapeutic efficacy and potential toxicity.

Differences in the TME, even within the same cancer type, can shape how CD4^+^ T cells respond to immunotherapies [114]. In patients with metastatic castration-resistant prostate cancer, anti-CTLA-4 therapy induced a Th1 response in soft-tissue tumors but failed to do so in bone metastases, where CD4^+^ T cells instead polarized toward a Th17 phenotype. Analysis of patient bone marrow samples revealed that high TGF-β levels in bone metastases suppressed Th1 differentiation, contributing to resistance to anti-CTLA-4 therapy [114].

Chimeric antigen receptor (CAR) T-cell therapy has transformed the treatment of certain hematologic malignancies. In a landmark study, CD4^+^ CAR-T cells (CD19-targeted), rather than CD8^+^ CAR-T cells, emerged as the primary effectors maintaining remission in two patients with chronic lymphocytic leukemia for nearly a decade [115]. While the initial response involves CD8^+^ and γδ T cells, proliferative and cytotoxic CD4^+^ CAR-T cells ultimately dominate. These cells expressed cytotoxic markers (e.g., GZMA, GZMK, and PRF1), remained metabolically active, and responded robustly to CD19^+^ leukemia cells in vitro without signs of exhaustion. Over time, the CAR-T-cell repertoire became clonally restricted, dominated by stable CD4^+^ clones that sustained antitumor function [115].

**Fig. 5:**
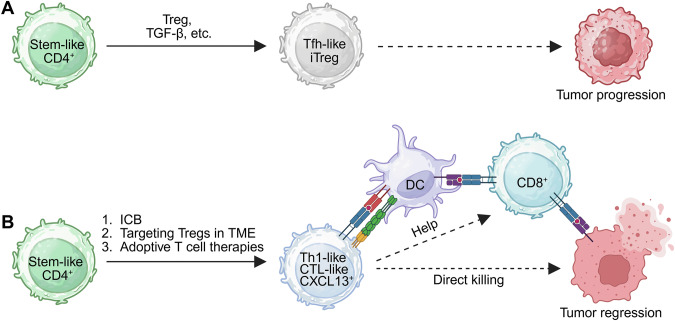
Functional fate decisions of stem-like CD4^+^ T cells determine tumor control outcomes. Stem-like CD4^+^ T cells in the tumor microenvironment (TME) can differentiate into either immunosuppressive or effector lineages, shaping tumor progression or regression. **A** In the presence of Tregs, TGF-β, and other suppressive cues, stem-like CD4^+^ T cells preferentially differentiate into Tfh-like or induced regulatory T cells (iTregs), leading to immune suppression and tumor progression. **B** Interventions such as immune checkpoint blockade (ICB) and preclinical strategies targeting intratumoral Tregs redirect stem-like CD4^+^ T cells toward Th1-like, cytotoxic (CTL-like), and CXCL13^+^ effector fates. These effector CD4^+^ T cells enhance dendritic cell (DC) function, support CD8^+^ T-cell responses, and can also directly kill tumor cells, ultimately promoting tumor regression. Adoptive T-cell therapies (e.g., CAR-T or TCR-T) using CD4^+^ T cells provide opportunities to genetically delete or enhance regulatory genes to favor effector differentiation. Other approaches, such as cancer vaccination or the use of oncolytic viruses that indirectly promote T-cell function, are not described here. Created with BioRender.com

Together, these findings demonstrate that the differentiation of CD4^+^ T cells into effector subsets, such as Th1-like, cytotoxic, and CXCL13^+^ Th cells, is critical for the success of cancer immunotherapies. This differentiation is influenced by the tumor type, microenvironmental signals, and nature of the immunotherapeutic approach. Strategies that increase the effector potential of stem-like CD4^+^ T cells while overcoming suppressive influences such as regulatory T (Treg) cells and immunosuppressive niches may be essential for achieving durable responses across a broad range of cancers (Fig. 5).

### In peripheral immune tolerance

In the periphery, self-reactive T cells that escape central tolerance are regulated through mechanisms such as anergy, deletion, and suppression by Treg cells to prevent autoimmunity [3, 4]. These tolerogenic mechanisms also extend to nonself antigens, including dietary components, commensal microbiota, and semiallogeneic fetal antigens during pregnancy, maintaining immune homeostasis across diverse contexts [14, 15, 116–118]. In these settings, CD4^+^ T cells encounter their cognate antigens, yet their differentiation is directed away from inflammatory effector fates (Fig. 4). This raises two key questions: (1) Do early antigen-primed CD4^+^ T cells in tolerogenic environments represent a distinct form of stem-like CD4^+^ T cells? (2) What factors drive early primed CD4^+^ T cells toward tolerogenic fates?

Studies in murine models have demonstrated that CD4^+^ T cells can undergo peripheral clonal deletion or exhaustion under conditions of high antigen load [88, 119]. For example, when OVA-specific CD4^+^ T cells are transferred into mice systemically expressing OVA, the cells initially proliferate but eventually undergo apoptosis, resulting in peripheral clonal deletion [119]. In contrast, when I-Eα-specific TEa cells are transferred into CB6F1 mice expressing I-Eα on antigen-presenting cells, the TEa cells persist but gradually acquire an exhausted phenotype [88]. Notably, in both settings, a subset of CD4^+^ T cells retains a TCF1^+^ stem-like phenotype before deletion or exhaustion occurs. Strikingly, when these early primed TCF1^+^ T cells are transferred into new hosts, they effectively mediate the rejection of OVA- or I-Eα-expressing skin grafts (unpublished observations). These findings underscore the role of environmental cues in shaping the fate of stem-like CD4^+^ T cells: while they resist effector differentiation under tolerogenic conditions, they retain the capacity to drive immune responses when the context shifts.

The role of Treg suppression in peripheral immune tolerance has been well established [4]. Interestingly, a study investigated the mechanisms of tolerance to tissue-restricted self-antigens using a model antigen (Cre recombinase) expressed in different tissues of transgenic mice [120]. Antigen-specific CD4^+^ T cells were tracked via tetramer-based enrichment, revealing that deletional tolerance was largely absent for these antigens. Instead, tolerance is maintained by thymically derived, antigen-specific Treg cells, particularly in the lung and intestine. However, Treg-mediated suppression was reversible, as successive antigen challenges (Listeria-Cre infection followed by rechallenge with Cre peptide + CFA) disrupted tolerance [120]. These findings indicate that Treg-mediated tolerance is dynamic and can be lost under inflammatory conditions. It remains to be determined how stem-like CD4^+^ T cells behave under sustained Treg suppression and how they retain the capacity to differentiate into effector cells when suppression is lifted.

To maintain intestinal health, the immune system must strike a delicate balance: effectively eliminating pathogenic microbes while preventing inappropriate immune responses to self-molecules, the commensal microbiota, and dietary components. Treg cells are central to this process, as they suppress excessive immune activation and promote tolerance to harmless antigens [116–118]. Studies have shown that manipulating the gut microbiota with Treg-inducing bacterial strains can alleviate diseases such as colitis and allergic diarrhea in experimental models [116–118]. Furthermore, research using OVA as a food antigen has demonstrated that oral tolerance is dependent on Treg cells. These Treg cells can be induced de novo in gut DLNs, migrate to intestinal tissues, and expand locally under the influence of intestinal macrophages [121].

A recent study tracked the fate of food antigen-specific CD4^+^ T cells before their differentiation into Treg cells [14]. Using the natural food antigen gliadin peptide (GLP) and the model antigen 2 W, the study revealed that following oral antigen exposure, food-specific CD4^+^ T cells expanded weakly in secondary lymphoid organs of the gut‒liver axis, primarily through Treg-mediated suppression. Most of these expanded cells lack canonical Th lineage markers and are classified as Th^lin‒^ cells, which are characterized by an anergic phenotype and resistance to Th1 differentiation. Over time, many Th^lin‒^ cells are converted into Tregs through an IL-2-dependent mechanism. In Treg-depleted mice fed 2 W peptides, Th^lin‒^, Tfh, and Th1 cells expanded 2-, 20-, and 100-fold, respectively [14]. These findings suggest that anergic Th^lin‒^ cells represent a subset of stem-like CD4^+^ T cells that serve as Treg precursors while retaining the potential to differentiate into Th1 cells when Treg suppression is removed or under inflammatory conditions.

The mechanisms by which maternal CD4^+^ T cells tolerate semiallogeneic fetal antigens during pregnancy remain unclear. A recent study investigated the fate of adoptively transferred OT-II cells in pregnant mice bearing mOVA transgenic fetuses [15]. In this model, B cells present mOVA antigen to OT-II cells, leading to their proliferation. However, sialylated glycans on trophoblast mOVA engage inhibitory receptors on B cells, activating CD22-LYN inhibitory signaling. This signaling limits costimulation, preventing OT-II cells from fully differentiating into IFN-γ^+^ effector cells [15]. Notably, another study demonstrated that mice previously exposed to mOVA during pregnancy can still reject OVA-expressing skin grafts [122]. Overall, the mOVA pregnancy model illustrates how, under tolerogenic conditions, antigen-specific CD4^+^ T cells proliferate without affecting effector differentiation but retain the capacity to differentiate into effectors when immune conditions change.

These studies highlight the dynamic regulation of CD4^+^ T cells in peripheral immune tolerance. While tolerogenic environments suppress effector differentiation, a subset maintains a stem-like state, preserving their potential for effector function when immune conditions shift. Further investigation is needed to define the signals that govern their fate and the mechanisms that balance tolerance and immune activation.

### Under immunosuppressive therapies

Immunosuppressive therapies are essential for suppressing transplant rejection and autoimmune diseases, yet they do not provide a cure. In transplantation, calcineurin inhibitors (CNIs), mTOR inhibitors, and antiproliferative agents reduce the risk of rejection but fail to eliminate alloreactive T cells, necessitating lifelong treatment [123]. Similarly, in autoimmune diseases, TNF inhibitors, JAK inhibitors, and IL-6 receptor antagonists suppress inflammation without inducing lasting immune tolerance [124]. A major challenge is the persistence of stem-like CD4^+^ T cells, which sustain immune responses in both settings [11, 12]. Elucidating how these cells evade immunosuppression is critical for developing more effective, targeted therapies that disrupt immune persistence at their source.

Calcineurin is essential for T-cell activation, as it is a protein phosphatase that dephosphorylates NFAT, enabling its nuclear translocation and initiation of gene transcription [125]. However, during persistent antigen stimulation, NFAT signaling also contributes to CD8^+^ T-cell exhaustion by inducing the expression of exhaustion-associated factors such as TOX and inhibitory receptors such as PD-1 [126–128]. Notably, tacrolimus, a CNI, suppresses TOX and PD-1 expression while upregulating TCF1 [127]. However, whether CNIs prevent exhaustion or merely delay its progression remains uncertain.

**Fig. 6:**
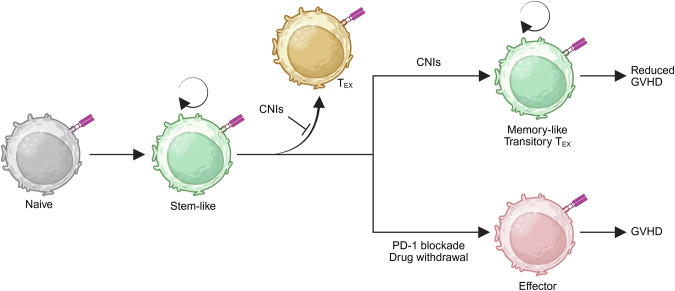
Stem-like T cells resist calcineurin inhibitor (CNI) therapies. In the context of allogeneic hematopoietic stem cell transplantation (HSCT), CNIs suppress the differentiation of stem-like T cells into both effector and terminally exhausted (T_EX_) cells. Instead, CNIs promote the persistence of memory-like T cells and transitory T_EX_ cells. This diversion away from terminal differentiation is associated with reduced GVHD during treatment. However, both memory-like and transitory T_EX_ cells retain their proliferative capacity and alloreactivity. Upon PD-1 blockade or CNI withdrawal, these cells can differentiate into pathogenic effector T cells, resulting in GVHD recurrence. Thus, while CNIs prevent terminal differentiation and exhaustion, they fail to eliminate the stem-like T-cell pool, highlighting their limitations in achieving durable immune tolerance. Created with BioRender.com

Two recent studies investigated how CNIs influence T-cell exhaustion and immune tolerance in murine allogeneic HSCT models [129, 130]. In one study, persistent alloantigen exposure following HSCT drove donor T cells toward terminal exhaustion (terminal T_EX_), characterized by high expression of PD-1, TOX, and TIGIT. However, cyclosporine treatment suppressed TOX expression and prevented terminal exhaustion, instead maintaining transitory exhausted T (transitory T_EX_) cells that expressed both inhibitory receptors and effector molecules. These transitory T_EX_ cells retain alloreactivity, respond to PD-1 blockade, and cause chronic GVHD after adoptive transfer into secondary recipients [129]. In the second study, both CNIs (cyclosporine and tacrolimus) preferentially expanded alloreactive CD4^+^ T cells after HSCT, despite persistent antigen stimulation. Rather than driving exhaustion, CNIs protected these CD4^+^ T cells in a TCF1^+^ T_CM_-like state, enabling self-renewal, proliferation, and resistance to apoptosis. Single-cell RNA sequencing and TCR analyses revealed that CNIs suppressed TOX and PD-1 expression, thereby preventing terminal exhaustion and instead maintaining a quiescent, T_CM_-like T-cell pool. While this effect reduces acute GVHD by restricting effector differentiation, it paradoxically sustains long-lived, alloreactive T cells, leading to chronic GVHD after drug withdrawal [130]. Together, these findings suggest that CNIs alone do not establish true immune tolerance, as they preserve alloreactive transitory T_EX_ and T_CM_-like cells rather than depleting them, underscoring the need for additional strategies to abolish pathogenic clones (Fig. 6).

mTOR plays a crucial role in regulating T-cell differentiation, balancing effector function and long-term persistence [131]. In CD8^+^ T cells, mTOR inhibition with rapamycin promotes memory T-cell generation in the context of acute viral infection or vaccination [132]. However, this effect was not observed in a skin transplant model [133]. During chronic infection or cancer, mTOR activity is required for the transition of progenitor exhausted T (T_PEX_) cells into terminally exhausted states, and its inhibition sustains the T_PEX_ pool, improving responses to immune checkpoint blockade [73].

These mechanisms are less studied in CD4^+^ T cells, but in an influenza infection model, rapamycin has been shown to regulate CD4^+^ T-cell fate by modulating the balance between stemness and exhaustion [83]. mTOR activity represses TCF1. By inhibiting mTOR signaling, rapamycin preserves TCF1 expression, maintaining a TCF1^+^ memory-like population while limiting terminal differentiation into Th1 effector cells [83]. Overall, rapamycin likely sustains a pool of self-renewing progenitors in both CD8^+^ and CD4^+^ T-cell responses, which may explain why rapamycin treatment disrupts effector differentiation but does not induce true immune tolerance in certain models.

These findings highlight the resistance of TCF1^+^ stem-like CD4^+^ T cells to immunosuppressive therapies. While CNIs and mTOR inhibitors suppress effector differentiation and acute immune responses, they do not eliminate self-renewing T-cell populations that sustain immune persistence. Instead, these therapies preserve TCF1^+^ progenitors, prevent terminal exhaustion, and maintain antigen-specific cells capable of reactivation upon drug withdrawal. Studies demonstrating T-cell resilience to CNIs and rapamycin in allogeneic HSCT and infections underscore the need to examine their effects in organ transplantation, where they are mainstay therapies. Additionally, understanding CD4^+^ T-cell resistance to other immunosuppressive therapies during transplantation and autoimmunity is crucial for disrupting immune persistence.

## Spatial and temporal adaptation of CD4^+^ T cells

CD4^+^ T cells exhibit remarkable plasticity, continuously adjusting their phenotypic and functional profiles in response to temporal changes and spatial cues within distinct tissue microenvironments [7, 134–139]. This process of clonal adaptation is shaped by antigen exposure, cytokine signals, and interactions with other cells, allowing CD4^+^ T cells to fine-tune their responses to pathogens, tumors, and self-antigens (Fig. 4). Recent advances in technologies, including single-cell RNA sequencing (scRNA-seq) combined with TCR sequencing (TCR-seq), have enabled high-resolution tracking of individual CD4^+^ T-cell clonotypes, revealing their gene expression profiles and dynamic adaptation across spatial and temporal contexts [7, 134–139].

A study analyzing 21 human cancer types via scRNA-seq revealed that CD4^+^ T cells in tumors commonly undergo differentiation into *IFNG*^+^ Tfh/Th1 dual-functional cells and *TNFRSF9*^+^ Treg cells [7]. The transition from *IL21*^+^ Tfh to Tfh/Th1 cells was marked by increased expression of *IFNG* and cytotoxic genes such as *GZMB* and *PRF1*, indicating the acquisition of effector function. *TNFRSF9*^+^ Treg cells emerge through a trajectory from the resting Treg state to the activated state, accompanied by increased interferon responses and the upregulation of transcription factors such as *HIVEP1*. These CD4^+^ T-cell changes are observed across cancer types and represent common differentiation pathways, although their abundance and prominence vary depending on the tumor type and microenvironmental cues, such as TGF-β signaling, type I interferons, and specific tumor mutations [7].

In a recent study, CD4^+^ T-cell clones were traced via paired scRNA/TCR-seq across tumors, tumor-adjacent normal tissues, lymph nodes, and peripheral blood from NSCLC patients treated with ICB [134]. The study revealed that Tfh and Treg cells displayed progressive transcriptional changes with increasing tumor proximity, resembling features of exhaustion. Despite having largely distinct TCR repertoires, Tfh and Treg cells share region-dependent gene expression programs, including the upregulation of exhaustion-associated genes such as *PDCD1*, *CTLA4*, and *TIGIT*. Notably, the authors identified a key finding: tumor-infiltrating Tfh cells were clonally linked to *TCF7*^+^ *PDCD1*^+^ stem-like progenitor populations residing in tumor-draining lymph nodes, suggesting a lineage relationship and a tissue-specific differentiation trajectory [134]. This study not only confirms and extends pancancer observations but also uniquely reveals a lymph node origin for stem-like CD4^+^ T cells that give rise to tumor-infiltrating Tfh cells, underscoring the importance of spatial and clonal relationships in shaping immune responses in cancer.

Another study traced CD4^+^ T-cell clones in colorectal cancer via paired scRNA/TCR-seq from tumors, adjacent normal tissue, and blood samples from 12 patients [135]. CD4^+^ T cells, including TEMRAs, Th17 cells, Tregs, and two distinct *IFNG*^+^ Th1-like subsets (GZMK^+^ vs. *CXCL13*^+^*BHLHE40*^+^), were found to occupy diverse functional states. TEMRA cells exhibited high clonal expansion and extensive TCR sharing across blood, normal mucosa, and tumors, indicating their migratory nature. In contrast, Th17 and *CXCL13*^+^*BHLHE40*^+^ Th1-like cells were clonally expanded and enriched specifically in tumors, with the latter being particularly abundant in microsatellite instability-high tumors. Most tumor-infiltrating Treg cells are clonally distinct, but some share TCRs with Th17 and Th1-like cells, suggesting the local induction of iTreg cells from helper subsets [135]. These findings highlight both migratory and tissue-adaptive clonal trajectories that shape the CD4^+^ T-cell landscape in colorectal cancer.

Paired scRNA/TCR-seq has been applied to identify neoantigen-specific CD4^+^ T-cell clones in metastatic cancers [136]. Seventeen CD4^+^ TCR clonotypes were experimentally validated by reconstructing TCRs in healthy donor T cells and testing their reactivity to patient-specific neoantigens via coculture assays with antigen-presenting cells and autologous tumor material. Most reactive TCRs originate from a transcriptionally distinct dysfunctional CD4^+^ T-cell cluster, marked by genes such as *CXCL13*, *PDCD1*, *TIGIT*, and *TOX*, and are clonally expanded within tumors. The authors also developed the NeoTCR4 gene signature, which enables the prediction of CD4^+^ neoantigen-specific T cells from scRNA-seq data without prior antigen knowledge [136]. This study provides a robust framework for discovering tumor-specific CD4^+^ TCRs and highlights their critical role in antitumor immunity.

To explore the long-term dynamics of antigen-specific CD4^+^ T-cell clones, a study used HLA-DQ:gluten tetramer-based sorting and high-throughput TCR sequencing to track gluten-specific CD4^+^ T cells in patients with celiac disease [137]. The authors analyzed matched blood and gut biopsy samples from 21 patients, with longitudinal sampling spanning from weeks to over 25 years, including before and after initiation of a gluten-free diet and during controlled oral gluten challenges. They reported that gluten-specific CD4^+^ T-cell clonotypes were remarkably stable over time, with up to 53% clonal overlap in samples taken more than two decades apart. Upon gluten re-exposure, the immune response was dominated by rapid expansion of preexisting memory T-cell clones rather than recruitment of new clones. Moreover, approximately 10% of TCR sequences are shared across individuals, reflecting a public and stereotyped T-cell response to gluten [137]. These findings demonstrate the persistence and recall potential of disease-driving CD4^+^ T cells in a human autoimmune setting and suggest that such clones could serve as biomarkers or therapeutic targets.

Another study investigated the temporal adaptation of autoantigen-specific CD4^+^ T cells in neuromyelitis optica spectrum disorder (NMOSD), where the key autoantigen is aquaporin-4 (AQP4) [138]. Using HLA class II tetramer staining, antigen-reactive T-cell enrichment, and single-cell RNA and TCR sequencing, the authors tracked AQP4-specific CD4^+^ T cells in patients over time. In newly diagnosed or untreated patients, these T cells show signs of activation and cytokine production (e.g., IFN-γ). However, in chronically treated individuals, these cells progressively acquired a stable exhaustion-like phenotype (ThEx), marked by PD-1, CTLA-4, TIGIT, and FOXP3 expression, and reduced effector function. These ThEx cells clonally expand, persist for up to four years, and retain AQP4 specificity [138]. These findings reveal a trajectory of temporal clonal adaptation, where persistent self-antigen exposure drives long-term maintenance of an exhausted yet autoreactive CD4^+^ T-cell pool, potentially contributing to disease chronicity and relapses in NMOSD.

In a murine model of retroviral infection, the authors used a TCRβ-transgenic system (EF4.1 mice) with a semipolyclonal CD4^+^ T-cell repertoire (diverse TCRα chains) to investigate how antigen-specific clonotypes adapt over time [139]. Following Friend virus infection, adoptively transferred CD4^+^ T cells mount an initial response dominated by high-avidity clones. However, as the infection progressed, the repertoire gradually diversified through the delayed and asynchronous recruitment of lower-avidity clonotypes, reflecting a process of temporal clonal adaptation. This late-phase diversification was dependent on antigen presentation by activated B cells via MHC class II but did not require germinal center formation or Tfh differentiation. Notably, B-cell-mediated antigen presentation relieves early interclonal competition, allowing expansion of lower-avidity clones that would otherwise be outcompeted [139]. This study demonstrated that B cells play an active and time-dependent role in shaping the breadth of the CD4^+^ T-cell response.

Together, these studies underscore the dynamic nature of CD4^+^ T-cell responses, which are shaped by both spatial and temporal cues that drive clonal expansion, differentiation, and adaptation [7, 134–139]. Advances in single-cell technologies and clonal tracking have revealed that CD4^+^ T-cell clonotypes migrate across tissues and undergo tissue-specific, time-dependent changes in phenotype and function driven by persistent antigen exposure, evolving tissue microenvironments, and cellular interactions [7, 134–139]. This emerging view of clonal adaptation offers key insights into how CD4^+^ T cells sustain immune surveillance, contribute to disease persistence, and respond to therapies across cancer, infection, and autoimmunity (Fig. 4).

## Therapeutic implications

### Reprogramming T-cell clonal adaptation for effective tumor control

Successful ICB therapies have been shown to drive stem-like CD4^+^ T cells toward effector fates [110–112]. However, in nonresponders, this transition is often impaired or dysregulated. Broadening the therapeutic benefit to more cancer patients will require strategies that selectively promote this transition while minimizing immune-related adverse events. Such strategies may involve overcoming the immunosuppressive tumor microenvironment (TME) or engineering T cells with greater resilience for use in ACT or CAR-T-cell therapies (Fig. 5).

Tumor-infiltrating Treg (TI-Tregs) play a central role in suppressing antitumor immunity and represent a major barrier to effective immunotherapy [140]. Notably, TI-Tregs differ from conventional Tregs in terms of phenotype, metabolic adaptations, and dependence on specific cytokines and surface molecules. These distinct features offer opportunities to selectively deplete or functionally impair TI-Tregs while preserving systemic immune tolerance [140]. In addition to TI-Tregs, other cell types within the TME, including cancer cells, endothelial cells, fibroblasts, and myeloid cells, collectively contribute to T-cell dysfunction through both intrinsic and paracrine immunosuppressive mechanisms [9]. A deeper mechanistic understanding of TME composition and plasticity is critical to developing precise and safe strategies that enable durable immune responses against cancer.

The application of CAR-T-cell therapies to solid tumors presents unique challenges that are now at the forefront of research [141, 142]. To address these hurdles, ongoing efforts are focused on identifying reliable surface markers selectively expressed on tumor cells and enhancing CAR-T-cell infiltration, persistence, and function within the TME. Emerging strategies include fine-tuning CAR signaling strength, improving T-cell fitness, and integrating safety switches or controllable systems to mitigate toxicity. Together, these advances are helping to expand CAR-T-cell therapy from a breakthrough in hematologic malignancies to a promising approach for treating solid tumors [141, 142].

TCR-engineered T cells used in ACT can target intracellular tumor antigens presented on MHC molecules, greatly expanding the pool of targetable antigens compared with the CAR-T-cell approach [143]. While tumor-infiltrating lymphocytes contain TCRs specific for tumor neoantigens, they often exhibit dysfunctional transcriptional signatures. By deciphering these signatures, researchers can predict and isolate neoantigen-specific TCRs directly from patient tumors [136]. As TCR discovery and engineering technologies continue to advance, neoantigen-directed TCR therapies are emerging as promising forms of precision immunotherapy for solid tumors.

T cells in tumors often undergo clonal adaptation toward dysfunctional states [125], posing a major barrier to the efficacy of both TCR-engineered and CAR-T-cell therapies. To overcome this challenge, a range of experimental strategies are being explored to reprogram therapeutic T cells with the goals of promoting stemness, supporting effector differentiation, enhancing resistance to dysfunction, and sustaining activity within the immunosuppressive TME. These approaches involve the overexpression or deletion of genes that regulate key aspects of T-cell biology, including transcription factors (e.g., IRF4, BATF, FOXO1, TBET, ETS1, and RBPJ), metabolic enzymes (e.g., Fdft1 and PDSS2), epigenetic modifiers (e.g., TET2 and ASXL1), and RNA-binding proteins (e.g., Regnase-1 and Roquin) [8, 144–155].

While many of these reprogramming strategies have been investigated in CD8^+^ T cells, their impact on antitumor CD4^+^ T cells remains relatively underexplored. Notably, unlike CD8^+^ T cells, which often undergo terminal exhaustion with the progressive loss of effector function, CD4^+^ T cells in tumors tend to become arrested in less differentiated, Tfh-like dysfunctional states [7, 125]. It is therefore critical to continue investigating whether these reprogramming approaches can redirect the clonal adaptation of CD4^+^ T cells, promoting their differentiation into effector and cytotoxic fates within the TME (Fig. 5).

### Targeting stem-like CD4^+^ T cells in autoimmunity and transplantation

In both autoimmunity and alloimmunity, self- or alloantigen-specific stem-like CD4^+^ cells serve as long-lived reservoirs that continually give rise to pathogenic effector populations [11, 12]. This persistent source of effector generation contributes to disease progression and resistance to immunosuppressive therapies [11, 12, 130]. Therefore, targeting stem-like CD4^+^ T cells represents a promising new strategy to eliminate the source of effector cells and promote tolerance induction.

However, significant challenges remain in the selective elimination of stem-like CD4^+^ T cells. Stem-like CD4^+^ T cells retain transcriptional and epigenetic features that closely resemble those of naïve and T_CM_ cells, which are critical for protective immunity against infections. For example, TCF1, a transcription factor essential for maintaining stem-like T cells, is also required for the development and maintenance of T_CM_ cells [11, 156]. Furthermore, TCF1 supports stemness by restraining effector differentiation. Although TCF1 deletion reduces the frequency of stem-like CD4^+^ T cells, it does not prevent the remaining stem-like cells from differentiating into effector cells [11]. In fact, in CTLA4-Ig‒treated mice, TCF1 deletion can accelerate acute transplant rejection rather than prevent it (unpublished observations).

Epigenetic regulation plays a key role in orchestrating the transition from stem-like to effector states by silencing genes associated with stemness and activating effector gene programs [157]. In parallel, stem-like CD4^+^ T cells undergo substantial metabolic reprogramming as they differentiate. Single-cell RNA-seq–based metabolic prediction has identified 1,497 metabolic reactions across 79 subsystems that differ significantly between stem-like and effector CD4^+^ T cells, with the vast majority being more active in effector cells [11]. These observations underscore the tight coordination between epigenetic and metabolic programs in driving effector differentiation.

Targeting these epigenetic and metabolic pathways offers a potential approach to block the transition of stem-like CD4^+^ T cells into pathogenic effector states [11]. However, it remains unclear whether such interventions merely preserve the stem-like pool or can redirect these cells toward alternative, nonpathogenic fates. Given that iTreg differentiation and dysfunction represent adaptive responses to chronic antigen stimulation [14, 121], it is plausible that with the right interventions, stem-like CD4^+^ T cells could be steered away from effector differentiation and instead pushed toward dysfunctional or tolerogenic states. Identifying such epigenetic and metabolic strategies may provide durable immune regulation in both autoimmunity and transplantation.

Although stem-like CD4^+^ T cells that emerge early after activation retain surface markers typically associated with naïve T cells, prolonged antigen exposure or tolerogenic therapies can induce the de novo expression of additional receptors [158, 159]. In an islet transplant tolerance model, where donor-specific tolerance was established by infusion of ethylene carbodiimide-fixed donor splenocytes, a population of CD73^+^FR4^+^ anergic CD4^+^ T cells was found to persist in tolerized recipients [158]. Upon MCMV infection, these anergic cells regain effector function and produce IFN-γ, contributing to allograft rejection. Importantly, targeted depletion of this population via the use of anti-FR4 antibodies prior to infection preserved transplant tolerance [158]. Thus, while stem-like CD4^+^ T cells can give rise to anergic populations with the potential for reactivation, anergic markers such as FR4 can be targeted to eliminate alloreactive cells and maintain durable immune tolerance.

Similarly, PD-1 expression is a hallmark of activated T cells and is markedly upregulated on stem-like T_PEX_ cells and terminally exhausted T cells under chronic antigen exposure [66]. A recent study using PD-1–specific depleting antibodies demonstrated that targeted depletion of PD-1^+^ cells effectively prevented heart transplant rejection and attenuated EAE [159]. These findings support the therapeutic potential of depleting persistence-prone T-cell subsets on the basis of surface receptor expression [158, 159], offering a strategy to enforce peripheral tolerance by eliminating stem-like T cells and their progeny in autoimmunity and transplantation.

Stem-like CD4^+^ T cells are vulnerable to chronic antigen exposure, which results in sustained proliferative pressure and cellular stress. Unlike naïve or T_CM_ cells, which remain largely quiescent, antigen-specific stem-like CD4^+^ T cells in autoimmunity and transplantation are in a state of ongoing activation, continuously cycling to maintain self-renewal and generate effector progeny [11, 12]. This persistent proliferation renders these cells uniquely susceptible to replication stress and genomic instability, suggesting a potential point of therapeutic intervention.

A seminal study identified the DNA base excision repair enzyme Apex1 as a critical regulator of T-cell fate under these conditions [160]. Apex1 is indispensable for repairing apurinic/apyrimidinic (A/P) DNA lesions that accumulate during rapid T-cell proliferation, acting as an essential checkpoint that safeguards genomic integrity. Conditional deletion of Apex1 in T cells protects mice from multiple autoimmune and allergic disease models, including EAE, allergic airway inflammation, and lupus-like disease [160]. In all cases, *Apex1*-deficient T cells fail to differentiate into effector subsets (e.g., Th1, Th2, and Th17), produce inflammatory cytokines, or infiltrate target tissues. Notably, pharmacologic inhibition of Apex1’s base repair activity with methoxyamine hydrochloride recapitulated these protective effects, even when Apex1 was administered after disease onset, underscoring its therapeutic potential [160]. Importantly, as dividing T cells accumulate large numbers of A/P DNA lesions that must be repaired in a timely fashion to prevent cell death, targeting genomic instability pathways may hold promise in limiting T-cell stemness in the treatment of transplant rejection and autoimmune diseases.

**Fig. 7:**
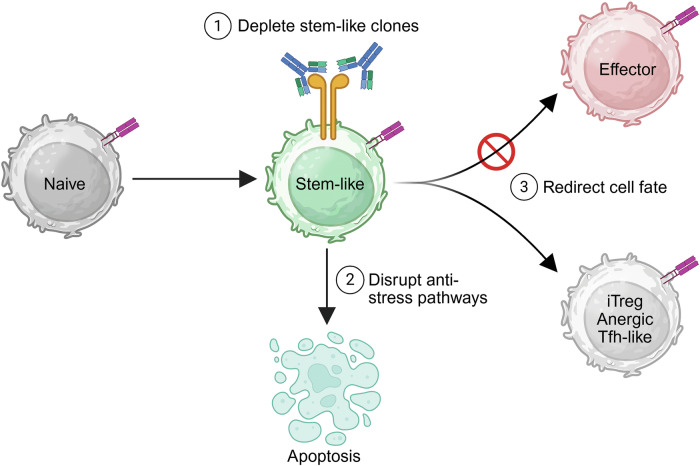
Strategies to target stem-like CD4^+^ T cells for durable immune tolerance. Stem-like CD4^+^ T cells serve as progenitors of pathogenic effector cells in autoimmune and alloimmune diseases. Targeting these cells through elimination or reprogramming may enable long-lasting immune control. Three potential approaches are illustrated: (1) Selective depletion of stem-like clones by identifying unique surface markers; (2) disruption of antistress pathways that sustain their self-renewal capacity, thereby promoting apoptosis; and (3) redirection of cell fate by modulating epigenetic or metabolic programs to promote differentiation into nonpathogenic states, such as iTregs, anergic cells, or Tfh-like cells, while blocking effector differentiation. Created with BioRender.com

Overall, uncovering the inherent vulnerabilities of stem-like CD4^+^ T cells may be key to effectively targeting them in autoimmunity and alloimmunity. Future studies are needed to (1) identify distinct surface receptors that enable selective depletion; (2) elucidate antistress mechanisms that preserve their self-renewal capacity, which could be disrupted to induce apoptosis; and (3) explore epigenetic and metabolic programs that may be manipulated to divert their differentiation toward nonpathogenic or tolerogenic fates (Fig. 7). These strategies may ultimately allow for durable immune regulation by eliminating or reprogramming the progenitors of pathogenic effector responses.

## Concluding remarks and perspectives

The identification of stem-like CD4^+^ T cells has introduced a new dimension of T-cell biology, especially in explaining the persistence of immune responses. These cells possess the capacity for self-renewal and effector differentiation, acting as reservoirs to sustain effector responses in autoimmunity, allergy, transplantation, and chronic infection [11–13, 16, 86, 90]. Conversely, under cancer and tolerogenic conditions, their failure to give rise to functional effectors contributes to immune dysfunction or tolerance [8, 14, 15]. Rather than reflecting *stochastic* flexibility, their fate decisions are shaped by clonal adaptation, an integrated and *deterministic* process in which antigen exposure, tissue-specific cues, and transcriptional–metabolic programs converge to guide distinct functional outcomes.

Importantly, the concept of stemness helps resolve a long-standing paradox: the persistence of T-cell responses during transplantation and autoimmunity despite chronic immunosuppression. Emerging evidence suggests that conventional immunosuppressive therapies often impair effector cells while sparing stem-like cells, which retain the capacity to reignite pathogenic responses [129, 130]. As such, durable immune tolerance may require strategies that specifically target stem-like CD4^+^ T cells. For example, disrupting the mechanisms and pathways that preserve their self-renewal capacity could render these cells vulnerable, enabling the selective elimination of their stemness and the induction of apoptosis in otherwise persistent clones [160].

The same principle can be leveraged in cancer immunotherapy, where enhancing the effector differentiation of stem-like CD4^+^ T cells may help overcome immunotherapy resistance. Genetic engineering approaches, such as modulating transcriptional regulators such as IRF4 or rewiring metabolic pathways, are being explored to sustain stemness while promoting tumor infiltration and functional maturation of effector T cells [8, 144–155].

Despite these challenges, continued efforts to decode the stress‒response circuits that preserve T-cell stemness and to map the rules that govern clonal adaptation will be critical for advancing therapeutic innovation. Emerging technologies, including single-cell multiomics, CRISPR-based perturbations [161], and high-resolution clonal tracking, promise to accelerate this effort, enabling precision interventions tailored to specific disease contexts.
